# MDR and XDR typhoid fever-a threat for the current decade?

**DOI:** 10.11604/pamj.2021.38.403.29244

**Published:** 2021-04-27

**Authors:** Manas Pustake, Mohammad Arfat Ganiyani, Girish Shakuntal

**Affiliations:** 1 Department of Internal Medicine, Grant Government Medical College and Sir JJ Group of Hospitals, Byculla, Mumbai, 400008, India,; 2 Department of Community Medicine, Grant Government Medical College and Sir JJ Group of Hospitals, Byculla, Mumbai 400008, India,; 3 Department of Pediatrics, SMBT Institute of Medical Sciences and Research Center, Dhamangaon, Nashik, Maharashtra, 422403, India

**Keywords:** Extensively drug-resistant typhoid fever, multidrug-resistant typhoid fever, Salmonella

## To the editors of the Pan African Medical Journal

Salmonella typhi is a gram-negative, flagellated, rod-shaped bacterium that is responsible for typhoid fever. Salmonella typhi infections are found only in humans and are spread by polluted water sources and poor hygiene practices, such as fecal contamination of edibles, insufficient hand washing, and so on. Despite significant advancements in health care delivery and medicine, millions of people around the world are at risk of contracting typhoid and paratyphoid fever as a result of exposure to the causative organism, which can result in disabilities and even death [1]. The African continent has had the world's highest case fatality rate and the longest median period of typhoid fever [2]. The World Health Organization in 2010 projected that the global burden of foodborne diseases was 33 million disability-adjusted life years (DALYs). Further to that, Africa has the largest incidence of food-borne diseases per household [3]. Inadequate sanitary practices have also contributed to an uptick in typhoid outbreaks following the COVID-19 pandemic. Previously, the discovery of new drugs to treat typhoid fever saved millions of lives all over the world. Unfortunately, decades of antibiotic use have resulted in the development of multidrug-resistant and extensively drug-resistant Salmonella typhi strains. In the case of typhoid fever, multidrug resistance (MDR) stands for resistance to Ampicillin, Trimethoprim-Sulfamethoxazole, and Chloramphenicol, whereas Extensive drug resistance (XDR) stands for Chloramphenicol, Ampicillin, Trimethoprim-Sulfamethoxazole, Fluoroquinolones, and third-generation Cephalosporin resistance.

**Figure 1:**
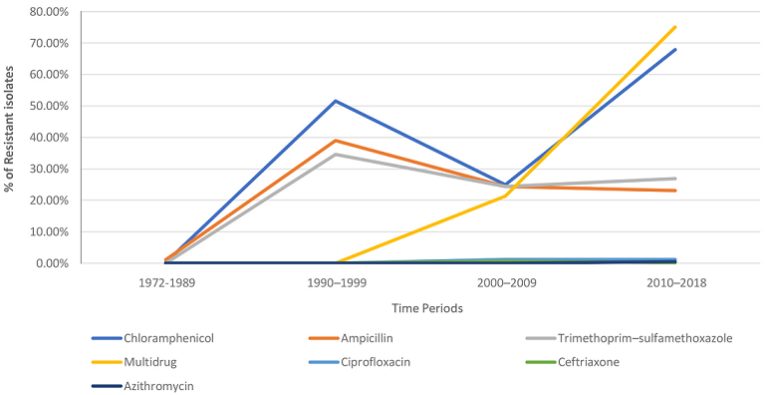
proportion of antimicrobial resistance in *S. Typhi* in Africa in the last four decades

In comparison to Asia, resistance to the three conventional first-line antimicrobials and multi-drug resistance occurred much later in Africa. The MDR H58 haplotype, which is known for its ability to spread globally and displace endemic Salmonella typhi, is thought to be introduced to Africa from Asia. Phylogenetic research backs this up as well. MDR strains are now presenting a major health danger in the African continent. Between 2010 and 2018, the prevalence of MDR strains on the continent rose from 0% to 38%. Resistance to ceftriaxone, ciprofloxacin, and azithromycin has also gained prominence in recent decades in the continent [4] (Figure 1). While no cases of XDR typhoid have been identified in Africa to date [4], the continent is already on the brink of being introduced to the organism's XDR variants. Since most African Low-Income Countries (LIC) and Low-Middle Income Countries (LMIC), particularly those where MDR typhoid has already been reported, depend entirely on clinical signs and titers and rarely conduct Antibiotic Susceptibility Testing (AST) due to financial constraints, this loophole in the national surveillance systems can hamper early reporting of cases [5]. On top of that, if the spread of MDR *S. typhi* is anything to go by, the increase of XDR strains in Asia could be a warning sign for Africa to be prepared for combating the XDR strains.

Several campaigns were launched throughout the continent to collect data related to the epidemiology of the disease. The Typhoid Surveillance in Africa Program (TSAP) that started in 2009 reported that the observed average burden of typhoid fever in Africa was two to three times higher than previously thought. Furthermore, almost half of all isolates are resistant to first-line antimicrobials [6]. In 2016, the severe typhoid fever in Africa (SETA) study was launched to gather comprehensive data on the clinical and financial burden of typhoid disease and its sequelae, as well as to characterize antimicrobial resistance patterns (AMR) [7]. The findings of these studies can be crucial for government officials when they prepare and implement public health strategies related to the disease (Figure 1). Chloramphenicol, Trimethoprim-Sulfamethoxazole, Ampicillin, Ciprofloxacin, Ceftriaxone, Azithromycin, Meropenem, and Tigecycline are currently recommended antibiotics for typhoid fever [8]. The last three drugs are usually used only for the XDR cases. Recently resistance has also been observed to Azithromycin [9], which leaves Meropenem and Tigecycline as the only alternatives, posing a significant threat to the outpatient management of the disease. Given that these are parenteral antibiotic formulations, the overall cost of the therapy would be beyond the reach of many African LICs and LMICs. Several case studies from Africa and Asia show that extreme weather conditions, such as flooding, may increase the likelihood of infectious diseases transmitting across water systems, as previously documented by the spread of various food-borne and water-borne illnesses [10]. This suggests that preventive interventions are needed to avoid the entry and spread of XDR typhoid fever throughout the continent.

## Conclusion

Community hygiene, sanitary measures, vaccination, treatment of carriers, and effective oral antibiotics for disease management are all needed to reduce the existing public health burden of MDR typhoid across the continent. Furthermore, the severity of the threat must be recognized by health care authorities ahead of time for the appropriate countermeasures to be implemented. There is an urgent need to develop an effective oral antibiotic against XDR typhoid and to implement preventive measures on a war footing to reduce the disease's health and financial burden, especially in the African continent where the disease is prevalent.

## References

[1] GBD 2017 Typhoid and Paratyphoid Collaborators (2019). The global burden of typhoid and paratyphoid fevers: a systematic analysis for the Global Burden of Disease Study 2017. Lancet Infect Dis.

[2] Appiah GD, Chung A, Bentsi-Enchill AD, Kim S, Crump JA, Mogasale V (2020). Typhoid outbreaks, 1989-2018: implications for prevention and control. Am J Trop Med Hyg.

[3] WHO WHO estimates of the global burden of foodborne diseases.

[4] Marchello CS, Carr SD, Crump JA (2020). A systematic review on antimicrobial resistance among *Salmonella Typhi* worldwide. Am J Trop Med Hyg.

[5] Lalremruata R, Chadha S, Bhalla P (2014). Retrospective audit of the widal test for diagnosis of typhoid Fever in pediatric patients in an endemic region. J Clin Diagn Res JCDR.

[6] Marks F, von Kalckreuth V, Aaby P, Adu-Sarkodie Y, El Tayeb MA, Ali M (2017). Incidence of invasive salmonella disease in sub-Saharan Africa: a multicentre population-based surveillance study. Lancet Glob Health.

[7] Park SE, Toy T, Cruz Espinoza LM, Panzner U, Mogeni OD, Im J (2019). The severe typhoid fever in Africa program: study design and methodology to assess disease severity, host immunity, and carriage associated with invasive salmonellosis. Clin Infect Dis Off Publ Infect Dis Soc Am.

[8] Dyson ZA, Klemm EJ, Palmer S, Dougan G (2019). Antibiotic resistance and typhoid. Clin Infect Dis.

[9] Hooda Y, Sajib MSI, Rahman H, Luby SP, Bondy-Denomy J, Santosham M (2019). Molecular mechanism of azithromycin resistance among typhoidal Salmonella strains in Bangladesh identified through passive pediatric surveillance. PLoS Negl Trop Dis.

[10] Cissé G, Traoré D, Touray S, Bâ H, Keïta M, Sy I (2016). Vulnerabilities of water and sanitation at households and community levels in face of climate variability and change: trends from historical climate time series in a West African medium-sized town. Int J Glob Environ Issues.

